# Equating patient-reported outcomes: developing a psychometric crosswalk between Scoliosis Quality of Life Index (SQLI) and Scoliosis Research Society (SRS-22r) to support long-term follow-up research in patients with adolescent idiopathic scoliosis

**DOI:** 10.1186/s41687-026-01087-0

**Published:** 2026-05-29

**Authors:** David Dueber, Henry Iwinski, Vishwas Talwalkar, Donna Oeffinger

**Affiliations:** 1 The Herb Innovation Center, University of Toledo, Toledo, USA; 2 Shriners Children’s Lexington, Lexington, USA

**Keywords:** Patient reported outcomes (PROS), Scoliosis Quality of Life Index (SQLI), Scoliosis Research Society (SRS-22r), Adolescent Idiopathic Scoliosis (AIS), Cross-walk linking

## Abstract

**Background:**

Despite widespread use of patient-reported outcome measures (PROs), research on measures in adolescent idiopathic scoliosis (AIS) is limited due to scarce large, long-term datasets. Our site began routinely collecting PRO data from patients with AIS in 2007, providing a robust longitudinal dataset. Initially, we used the adolescent-validated Scoliosis Quality of Life Index (SQLI) before transitioning to Scoliosis Research Society (SRS-22r) in 2010. Despite having over 2,400 pre-transition SQLI assessments, inability to convert SQLI to SRS-22r scores has limited our ability to use early data for long-term outcomes research. Recognizing this, we collected SQLI and SRS-22r concurrently, creating a unique opportunity to develop and validate a cross-walk between the two. The primary study aim is to establish such cross-walks, enabling the integration of historical SQLI data into contemporary longitudinal studies and meta-analyses.

**Methodology:**

Using a common persons linking design, this study analyzed SQLI and SRS-22r data from adolescents with idiopathic scoliosis collected during routine clinical care. Calibration and validation samples were formed from patients with concurrent administrations, supplemented by single-instrument visits. Assumptions for linking were evaluated through internal consistency, inter-measure correlations, confirmatory factor analysis, and sex-based invariance testing. Linear equating, equipercentile equating, and Rasch-based true score equating were evaluated using bias, RMSE, correlations, and Bland–Altman plots.

**Results:**

All domains met recommended criteria for linking, with strong correlations and low average errors. Linear equating consistently outperformed alternative methods, yielding minimal bias and stable error across score ranges. Linked scores demonstrated high agreement with observed SRS-22r scores for pain, self-image, and mental health, and moderate agreement for function. Linking of change scores resulted in somewhat lower correlations and higher mean error; however, bias remained small, suggesting that longitudinal group-level comparisons are supported.

**Conclusions:**

Crosswalks between historical SQLI and SRS-22r data were established, enabling their integration in long-term studies. While most domain linkages are strong, the function domain shows moderate quality. Aggregated transformed SQLI scores can reliably represent SRS-22r scores, allowing valuable historical data to inform treatment outcomes. This advancement enables more comprehensive longitudinal outcomes research by integrating patient-reported outcomes with clinical measures.

Adolescent Idiopathic Scoliosis (AIS) is a commonly treated problem in pediatric orthopedics. The use of validated patient reported outcome measures (PROs) to capture information directly from the patient’s perspective is helpful in quantifying how their health condition is affecting various aspects of their life, such as, function, mental health and overall quality of life. PROs are used routinely in clinical management and research to assess changes over time and outcomes of clinical treatments. Condition-specific, rather than generic PROs, can be more sensitive to change and better reflect the patient’s symptoms and functioning [1]. Early adopters of PROs started using them in the early 2000s, slowly moving the focus from research only use to application in clinical care. The Patient Protection and Affordable Care Act in 2010 expectation of hospital quality measurement accelerated the use of PROs into standard healthcare assessments.

Two validated condition specific PROs for individuals with AIS are the Scoliosis Quality of Life Index (SQLI) [2] and Scoliosis Research Society (SRS-22r) [3, 4]. The SRS-22 was developed to measure disease-specific health-related quality of life instrument for individuals with AIS undergoing posterior spinal fusion, and assesses five domains: pain, self-image/appearance, function/activity, mental health, and satisfaction with treatment [3, 4]. In 2005, the SQLI was developed and validated specifically for adolescents with AIS. This was done to address concerns that the original SRS-22, which was constructed and tested in an AIS population with a mean age of 25 years, might not be relevant to adolescents aged 10 to 18 years with scoliosis [2]. The SQLI modified the SRS-22 to focus directly on adolescents with AIS. It is a 22-item questionnaire with the domains of physical activity, back pain, self-esteem, moods & feelings and satisfaction with management. Since that time, the SRS-22 has undergone several modifications [5–8] and the SRS-22r is now the most commonly utilized health-related quality-of-life measure for adolescent idiopathic scoliosis [9, 10]and recommended as the primary PROs for inclusion in research involving patients with AIS [11]. Therefore, the SQLI is now considered a legacy measure and is infrequently used clinically or in research. In order for researchers to accurately compare results across studies using different quality of life PROs, incorporate studies using the SQLI and SRS-22r in a common meta-analysis, and allow investigators with historical SQLI data to incorporate that data in research studies, including long-term analyses of patients with AIS, the creation of a cross-walk table between SQLI and SRS-22r domain scores is necessary.

While the integration of PROs into clinical practices and research studies have greatly increased in recent years, research on the measures themselves has been limited. Few large, long-term data sets containing PROs for patients with AIS exist, thereby limiting the ability to conduct research to help improve PROs interpretability. Recognizing the importance of PROs in clinical care, our site began collecting PROs routinely from patients with AIS in 2007, thereby affording us a large longitudinal dataset available for analyses to help answer the questions about much-needed long-term outcome of care for patients with AIS. However, in the early days we opted to use the adolescent validated SQLI measure before transitioning to the SRS-22r in 2010. Our dataset includes three years and over 2400 SQLI assessments prior to the transition to routine administration of the SRS-22r. The inability to translate the SQLI scores to SRS-22r scores has limited our ability to capitalize on our early PRO clinical adoption to conduct the critically needed long-term outcome studies for patients with AIS. After the transition to routine collection of the SRS-22r, we continued to collect SQLI assessments at the same clinic appointment. We recognized that this data source put us in a unique position to conduct in-depth analyses to establish a cross-walk between the legacy measure, SQLI, and the currently accepted standard SRS PROM, the SRS-22r. Therefore, the primary purpose of this study was to establish these cross-walks and assess their validity. The creation of validated cross-walks will allow investigators who have historical SQLI data to incorporate it into their longitudinal studies and for historical studies that used SQLI to be included in modern meta-analyses.

## Methodology

This retrospective study analyzed data from individuals with a primary diagnosis of AIS (Cobb angle > 15degrees), who had clinically completed at least one SRS-22r between 4/28/2010 and 11/1/2021 and/or SQLI assessment between 10/6/2009 and 7/26/2017. After IRB approval, individual SRS-22r and SQLI question (item) scores and overall domain scores, and the date on which each assessment was completed, were retrieved from queries of the facility’s outcome databases and electronic medical record (EMR).

The study population was generated from a consecutive case review, including all eligible male and female patients and all ethnic backgrounds. Data from individuals with secondary diagnoses that could influence overall quality of life and/or answers on the SRS-22r assessment were excluded. Patient information collected through EMR review included diagnosis, age at assessment, sex, and race.

A total of 1528 patients completed assessments at 3477 visits. Of these patients, 559 completed both the SQLI and the SRS-22r within a single visit for a total of 853 visits. Additionally, 515 patients completed only the SQLI at a visit and 628 patients completed only the SRS-22r at a visit. From the visit data in which both SQLI and SRS-22r were completed, calibration (*N* = 384) and validation (*N* = 385) samples were created by randomly selecting one visit per patient for each sample; patients with only one visit were randomly assigned to either the calibration or validation sample. An extended calibration sample (*N* = 1528) was created by adding SRS-22r-only and SQLI-only visits (randomly selecting one per patient) to the calibration sample.

### Measures

The SQLI and SRS-22r questionnaires were completed by patients during routine clinic visits as part of standard clinical care and are administered to all patients with scoliosis who were at least 10 years of age. These instruments have been validated, and psychometric properties reported in the literature. SQLI is reliable and valid and demonstrates satisfactory distribution of scores [2]. For the SRS-22r, traditional psychometric properties of validity, reliability, and responsiveness have been widely studied and have shown it to have excellent convergent, discriminant, and construct validity for adolescents with AIS [12–21]. 

### SRS-22r

The SRS-22 [3, 17, 19, 22, 23] is a 22-item scoliosis specific health-related quality of life questionnaire. The SRS-22r includes 2 satisfaction items which are not considered in this study and 5 items in each of 4 domains: function, pain, self-image, and mental health. For each item, participants are asked to respond using an item-specific response scale with five options (lowest = 1, highest = 5); domain scores are the average of scores of the four items belonging to that domain. Sample internal consistency of domains ranges from ω = 0.72 (function) to ω = 0.87 (mental health).

### SQLI

The SQLI [2, 22–25] is a 22-item self-report scoliosis specific health-related quality of life questionnaire. The SQLI includes 2 satisfaction items which are not considered in this study and 5 items in each of 4 domains: physical activity, back pain, self-esteem, and mood and feelings. For each item, participants are asked to respond using an item-specific response scale with five options (lowest = 0, highest = 4); domain scores are the total of scores of the four items belonging to that domain. Sample internal consistency of domains ranges from ω = 0.79 (self-esteem) to ω = 0.86 (physical function).

### Assumptions

Linking of scores between different instruments requires that scores from both instruments demonstrate high (minimally α > 0.70 but ideally α > 0.80) and similar (αs within 0.10 of each other) internal consistency [26], scores from the two measures are highly correlated (minimally *r* > .70 but ideally *r* > .86); [27], the two instruments measure essentially the same construct, and the linking relationship is constant across subgroups. To test whether the two instruments measured essentially the same construct, a series of confirmatory factor analysis (CFA) models were fitted on the calibration sample and assessed. For each domain, items from both versions were combined and three models were fit using the lavaan package for R [28]. The first model was a unidimensional model in which all ten items load onto a single factor. The second model was a two-factor model in which SRS-22r and SQLI domain items loaded onto different factors. The SQLI and SRS-22r subdomains will be determined as measuring essentially the same construct if the difference in fit between the unidimensional and two-factor models is minimal, defined as a nonsignificant chi-square difference test or a change in Bayesian Information Criteria (BIC) of less than 10 [29]. To test for invariance of linking across subgroups, the standardized root expected mean square difference (REMSD) [30]; was computed for each linking function based on sex groups; other demographic groupings (race, ethnicity) were not considered due to insufficient sample size. Values of REMSD less than 8% were considered adequate evidence of subgroup invariance [26]. 

### Linking strategies

A variety of linking techniques were applied to the calibration or extended calibration sample to generate crosswalk tables between SQLI domain scores and SRS-22r domain scores. Linking of SQLI scores and SRS-22r scores was performed using linear equating on the calibration sample, equipercentile equating on the calibration sample, and a modified item response theory (IRT) true score equating on the extended calibration sample. Domain scores used throughout are within domain mean scores (average of 5 items; scores between 1 and 5) for SRS-22r domains and within domain total scores (total of 5 items; scores between 0 and 20). The results of these linking computations were evaluated on the validation sample; the best performing crosswalk table was reported. Linear equating and equipercentile equating were computed using the *equate* package for R [31]; polynomial log-linear smoothing was employed for equipercentile equating.

IRT true score equating was also performed using the mirt package for R [32, 33]; due to the relatively small sample size a Rasch partial credit model was fit to the data rather than an IRT graded response model. For each domain a unidimensional Rasch partial credit response model of all ten items (i.e., from both SRS-22r and SQLI) was fit to the extended calibration sample. Item parameters for the SQLI items were used to create a crosswalk between SQLI domain scores and Rasch measure scores. Item parameters for the SRS-22r items were used to transform Rasch measure scores into expected SRS-22r domain scores. This transformation enables creation of a crosswalk between SQLI domain scores and SRS-22r domain scores by converting SQLI domain scores to Rasch measure scores and then converting Rasch measure scores to SRS-22r domain scores.

Standard errors of linking were computed using bootstrapping for all equating methods using 1,000 bootstrap samples.

### Evaluation strategies

The accuracy of linking was assessed within the validation sample using several statistical indices. The first of these indices was the root mean square error (RMSE) of linking, defined as the root mean square of the differences between transformed SQLI domain scores and observed SRS-22r domain scores. The bias of linked scores, defined as the mean difference between transformed SQLI domain scores and observed SRS-22r domain scores, was also considered. Finally, the Pearson correlation between transformed SQLI domain scores and observed SRS-22r domain scores was considered.

Additionally, quality of linking will be evaluated qualitatively using Bland-Altman plots. Limits of agreement in the Bland-Altman plots are 1.96 standard deviations above and below the mean bias. While the statistical indices used to evaluate the quality of linking are all marginal (in the sense that a single score describes the entire score distribution), Bland-Altman plots can be used to evaluate the quality of linking conditional upon trait level.

Following selection of optimal linking function, the performance of linking was checked against change scores. Specifically, for each of the 420 patients who completed both the SRS-22r and the SQLI at multiple visits, a random pair of adjacent visits were chosen. Change scores for each domain were then computed based on observed SRS-22r domain scores and based on transformed SQLI domain scores. For each domain, RMSE, bias, and correlations were computed similarly as with individual domain scores.

## Results

The calibration sample included data from 384 individuals with a mean age of 14.9 ± 2.0 years, that was 83% female, and 91% white or of non-Hispanic ethnicity. The mean primary curve magnitude was 35⁰±13⁰ (range of 12⁰-75⁰) with a pattern distribution of 58% Thoracic, 25% Lumbar and 17% Thoracolumbar. The validation sample was very similar to this population.

The four SRS-22r subdomains (Function, Pain, Self-Image and Mental Health) and four SQLI subdomains (Self-Esteem, Back pain, Physical Activity and Moods & Feelings) exhibited omega estimates of internal consistency greater than 0.80 except SRS-22r function (ω = 0.72) and SQLI self-esteem (ω = 0.79). Internal consistency estimates for corresponding subdomains were very similar (difference less than 0.05) except for the SRS-22r function (ω = 0.72) and SQLI physical activity (ω = 0.86) domains. Correlations between matched subdomains were high, ranging from *r* = .76 (function) to *r* = .90 (pain). Fit results for CFA models checking the assumption that matching SRS-22r and SQLI subdomains measure the same construct can be found in Table 1. The two factor models do not significantly improve fit for the pain and mental health domains; for function and self-image, the improvement in model fit is very small as measured by BIC. In all two factor models, the correlation between SRS-22r and SQLI subdomains was greater than 0.90. Fit of the unidimensional models was quite poor, except for the function subdomain. Inspection of residual correlations revealed that the largest residual correlations were of SRS-22r and SQLI item pairs in which the items are nearly identical. For example, SRS-22r item 4 and SQLI item 1 are different phrasings of the same concept and even have the same response options. Because poor fit is largely explainable by pairs of items across instruments being overly similar, this poor fit was deemed to not be problematic for linking. The assumption of invariance of linking across subgroups was found to be tenable; REMSD < 0.08 for all linking techniques.

**Fig. 1:**
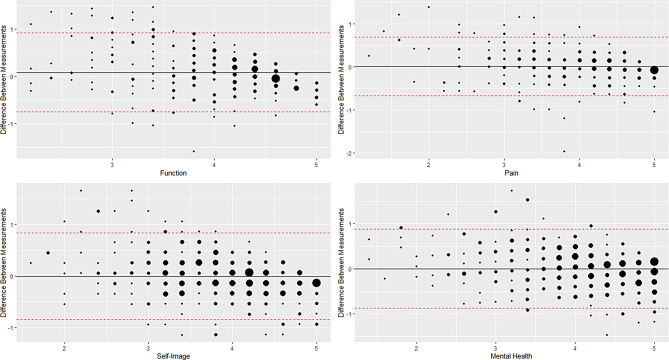
Bland Altman plots for linking SQLI domain scores to SRS-22r domain scores. SRS-22r: Scoliosis Research Society-22r; SQLI: Scoliosis Quality of Life Index

Linking was performed on the four subdomains using linear, equipercentile, and true score equating. Bias, RMSE, and correlations between linked and observed scores can be found in Table 2. Modified IRT true score equating was consistently the worst performing method. Linear and equipercentile equating resulted in very similar linking quality. The optimal equating method was chosen to be linear equating for all four domains due to having the less bias and comparable RMSE and correlations compared to the other methods. Correlations between transformed SQLI domain scores and SRS-22r domain scores were moderate for the function domain (*r* = .757) and high for the pain (*r* = .908), self-image (*r* = .818), and mental health (*r* = .842) domains. Crosswalk tables for these linking functions can be found in Table 3 and Bland-Altmann plots can be found in Fig. 1. Bland-Altmann plots show that, while variability between transformed SQLI domain scores and SRS-22r domain scores decreases as those scores increase, there remains a small chance of large (> 0.8) errors at all SRS-22r domain score levels. Because the optimal linking functions were linear, the transformation of SQLI domain scores into SRS-22r domain scores can be described by the following simple equations, where mean is the average of the 5 items within the domain:

$$\begin{aligned}&\mathrm{M}\mathrm{e}\mathrm{a}\mathrm{n}\:\mathrm{S}\mathrm{R}\mathrm{S}-22\mathrm{r}\:\mathrm{F}\mathrm{u}\mathrm{n}\mathrm{c}\mathrm{t}\mathrm{i}\mathrm{o}\mathrm{n}\cr &=\:(0.157\:\times\:\:\mathrm{S}\mathrm{Q}\mathrm{L}\mathrm{I}\:\mathrm{P}\mathrm{h}\mathrm{y}\mathrm{s}\mathrm{i}\mathrm{c}\mathrm{a}\mathrm{l}\:\mathrm{A}\mathrm{c}\mathrm{t}\mathrm{i}\mathrm{v}\mathrm{i}\mathrm{t}\mathrm{y})\hspace{0.17em}+\hspace{0.17em}1.733\end{aligned}$$

$$\begin{aligned}&\mathrm{M}\mathrm{e}\mathrm{a}\mathrm{n}\:\mathrm{S}\mathrm{R}\mathrm{S}-22\mathrm{r}\:\mathrm{P}\mathrm{a}\mathrm{i}\mathrm{n}\cr &=\:(0.193\:\times\:\:\mathrm{S}\mathrm{Q}\mathrm{L}\mathrm{I}\:\mathrm{B}\mathrm{a}\mathrm{c}\mathrm{k}\:\mathrm{P}\mathrm{a}\mathrm{i}\mathrm{n})\hspace{0.17em}+\hspace{0.17em}1.061\end{aligned}$$

$$\begin{aligned}&\mathrm{M}\mathrm{e}\mathrm{a}\mathrm{n}\:\mathrm{S}\mathrm{R}\mathrm{S}-22\mathrm{r}\:\mathrm{S}\mathrm{e}\mathrm{l}\mathrm{f}\:\mathrm{I}\mathrm{m}\mathrm{a}\mathrm{g}\mathrm{e}\cr &=\:(0.201\:\times\:\:\mathrm{S}\mathrm{Q}\mathrm{L}\mathrm{I}\:\mathrm{S}\mathrm{e}\mathrm{l}\mathrm{f}\:\mathrm{E}\mathrm{s}\mathrm{t}\mathrm{e}\mathrm{e}\mathrm{m})\hspace{0.17em}+\hspace{0.17em}0.844\end{aligned}$$

$$\begin{aligned}&\mathrm{M}\mathrm{e}\mathrm{a}\mathrm{n}\:\mathrm{S}\mathrm{R}\mathrm{S}-22\mathrm{r}\:\mathrm{M}\mathrm{e}\mathrm{n}\mathrm{t}\mathrm{a}\mathrm{l}\:\mathrm{H}\mathrm{e}\mathrm{a}\mathrm{l}\mathrm{t}\mathrm{h}\cr &=\:(0.222\:\times\:\:\mathrm{S}\mathrm{Q}\mathrm{L}\mathrm{I}\:\mathrm{M}\mathrm{o}\mathrm{o}\mathrm{d}\mathrm{s}\:\&\:\mathrm{F}\mathrm{e}\mathrm{e}\mathrm{l}\mathrm{i}\mathrm{n}\mathrm{g}\mathrm{s})\hspace{0.17em}+\hspace{0.17em}0.716\end{aligned}$$

Indices evaluating the accuracy of linear equating for change scores can be found in Table 4. Bias and RMSE were higher for change scores than for individual visit scores, but the average bias for all patients was less than 0.05 for all domains. Correlations between transformed SQLI domain change scores and SRS-22r domain change scores were low (i.e., less than 0.70) for the Function (*r* = .593) and Self-Image (*r* = .651) domains and moderate for the Pain (*r* = .789) and Mental Health (*r* = .706) domains.

**Table 1:** Fit indices for unidimensional confirmatory factor analysis models and fit comparison with two factor models

SRS-22r Domain	Unidimensional model						Two factor model		
	χ^2^	*p*	RMSEA	CFI	TLI	SRMR	Δχ^2^	Δp	ΔBIC
Function	χ^2^(35) = 112.76	< 0.001	0.076	0.951	0.937	0.042	χ ^2^(1) = 10.54	0.001	4.59
Pain	χ^2^(35) = 112.76	< 0.001	0.193	0.829	0.781	0.099	χ ^2^(1) = 0.34	0.557	5.61
Self-Image	χ^2^(35) = 477.91	< 0.001	0.182	0.796	0.738	0.097	χ ^2^(1) = 5.15	0.023	0.81
Mental Health	χ^2^(35) = 279.64	< 0.001	0.135	0.892	0.861	0.057	χ ^2^(1) = 2.07	0.150	3.88

Note. RMSEA = root mean square error of approximation, CFI = comparative fit index, TLI = Tucker-Lewis index, SRMR= standardized root mean squared residual, BIC = Bayesian information criterion. SRS-22r: Scoliosis Research Society-22r; SQLI: Scoliosis Quality of Life Index

### Clinical applications of results

Consistent with other linking studies with PROS [34, 35] using the crosswalk tables and linking functions should be used to convert scores for individual patients from SQLI domain scores to SRS-22r domain scores for inclusion in group analyses only. Use of converted scores for clinical management is not recommended. This is illustrated by a randomly selected patient from our sample who had a SQLI Moods & Feelings score of 18 and an SRS-22r Mental Health score of 4.2. The SQLI Moods & Feelings domain score can be converted using the crosswalk table or the formula to arrive at a mean SRS-22r Mental Health score of 4.71; this score differs notably from the observed mean SRS-22 Mental Health score of 4.2.

Use of the linking functions to assess groups of patients is supported by the study findings. When aggregating across many patients, the average error is very small thus supporting conversion of SQLI scores to SRS-22r scores. When investigators have access to individual patient scores, these scores should be converted and then used in aggregate. However, if only mean SQLI scores are available, these summary data can also be accurately converted using the linking functions.

For example, to compare changes between curve severity groups we used data from adjacent timepoints of patients with multiple assessments. Within our study sample, individual patient SQLI scores were converted to SRS-22r scores using the cross-walk table (Table 3). Average scores from each visit and change scores were compared between observed SRS-22r scores and linked SQLI scores (Table 4). For visit and change scores the mean difference between SRS-22r observed and linked scores was excellent (0.13 or less), illustrating the utility of this methodology for use in longitudinal studies. When group sample sizes are sufficiently large, such as the example groups where samples range from 82 to 244, use of the linking functions is supported.

Additionally, using the transformation equations to convert summary statistics from SQLI domains to SRS-22r domains is supported and could be applied in meta-analysis. As an example, we used the linking functions to convert mean SQLI Self Esteem scores to mean SRS-22r Self Image scores for males and females and compared the results to the actual mean SRS-22r Self Image scores. The male group (*N* = 69) in the validation sample has a mean SQLI Self Esteem score of 15.25. Using the linking equation, this mean score can be converted to a mean SRS-22r Self Image score of 15.25 × 0.201 + 0.844 = 3.91, which is close to the mean SRS-22r Self Image score of 3.97 for these patients. The female group (*N* = 316) in the validation sample has a mean SQLI Self Esteem score of 14.77. Using the linking equation, this mean score can be converted to a mean SRS-22r Self Image score of 14.77 × 0.201 + 0.844 = 3.81, which is very close to the observed mean SRS-22r Self Image score of 3.83 for these patients.

**Table 2:** Comparison of linking accuracy between linked and observed scores

	RMSE	Bias	Correlation
**SRS-22r Function**			
Linear Equating	0.434	0.082	0.757
Equipercentile Equating	0.424	0.089	0.760
True Score Equating	0.427	0.105	0.751
**SRS-22r Pain**			
Linear Equating	0.347	0.007	0.908
Equipercentile Equating	0.342	0.011	0.910
True Score Equating	0.351	0.037	0.907
**SRS-22r Self-Image**			
Linear Equating	0.429	0.006	0.818
Equipercentile Equating	0.429	0.013	0.818
True Score Equating	0.417	0.033	0.819
**SRS-22r Mental Health**			
Linear Equating	0.448	0.003	0.842
Equipercentile Equating	0.431	0.003	0.841
True Score Equating	0.432	0.029	0.843

Note. RMSE = root mean square error. SRS-22r: Scoliosis Research Society-22r

**Table 3:** Crosswalk table converting SQLI Subdomain and Total Scores to SRS-22r scores

SQLI Subdomain Total Score	SRS-22r Function Score (SE)	SRS-22r Pain Score (SE)	SRS-22r Self-Image Score (SE)	SRS-22r Mental Health Score (SE)
0	1.73 (0.21)			
1	1.89 (0.19)	1.25 (0.20)		0.94 (0.23)
2	2.05 (0.17)	1.45 (0.18)	1.25 (0.13)	1.16 (0.20)
3	2.20 (0.15)	1.64 (0.16)	1.45 (0.11)	1.38 (0.18)
4	2.36 (0.13)	1.83 (0.14)	1.65 (0.10)	1.60 (0.15)
5	2.52 (0.12)	2.03 (0.12)	1.85 (0.09)	1.83 (0.13)
6	2.67 (0.10)	2.22 (0.11)	2.05 (0.08)	2.05 (0.11)
7	2.83 (0.08)	2.41 (0.09)	2.25 (0.06)	2.27 (0.10)
8	2.96 (0.07)	2.61 (0.08)	2.45 (0.05)	2.49 (0.08)
9	3.14 (0.06)	2.80 (0.07)	2.65 (0.04)	2.71 (0.07)
10	3.30 (0.05)	2.99 (0.06)	2.85 (0.04)	2.94 (0.05)
11	3.46 (0.04)	3.19 (0.04)	3.05 (0.03)	3.16 (0.04)
12	3.61 (0.03)	3.38 (0.04)	3.26 (0.02)	3.38 (0.03)
13	3.77 (0.02)	3.57 (0.03)	3.46 (0.02)	3.60 (0.03)
14	3.92 (0.02)	3.76 (0.02)	3.66 (0.02)	3.82 (0.02)
15	4.08 (0.01)	3.96 (0.02)	3.86 (0.01)	4.04 (0.02)
16	4.24 (0.01)	4.15 (0.01)	4.06 (0.01)	4.27 (0.01)
17	4.39 (0.01)	4.34 (0.01)	4.26 (0.01)	4.49 (0.01)
18	4.55 (0.01)	4.54 (0.01)	4.46 (0.01)	4.71 (0.01)
19	4.71 (0.01)	4.73 (0.01)	4.66 (0.02)	4.93 (0.01)
20	4.86 (0.01)	4.92 (0.01)	4.86 (0.02)	5.15 (0.02)

Note. SE = standard error of equating. SRS-22r: Scoliosis Research Society-22r; SQLI: Scoliosis Quality of Life Index

**Table 4:** Accuracy of linear equating function for change scores

	RMSE	Bias	Correlation
SRS-22r Function	0.545	-0.085	0.593
SRS-22r Pain	0.456	-0.027	0.789
SRS-22r Self-Image	0.508	-0.023	0.651
SRS-22r Mental Health	0.476	-0.005	0.706

Note. RMSE = root mean square error. SRS-22r: Scoliosis Research Society-22r

## Discussion

Despite well-documented short-term outcomes in adolescent idiopathic scoliosis (AIS), research addressing the long-term psychosocial and functional impacts remains relatively scarce [36]. Most existing assessments predominantly rely on quantitative measures such as radiographic curvature or standardized health-related quality of life (HRQoL) questionnaires [36–39]. The lack of available long-term data from patient reported outcome measures limits the ability to link clinical measures to the impact on function and psychosocial well-being. Without PRO data valuable information about the experiences of patients over time is not captured, overlooking the important dimensions of their enduring well-being [39]. Studies that assess long-term outcomes of AIS treatments utilizing patient-reported outcomes (PROs) is essential to deepen our understanding of both the disease and its treatments from a truly patient-centered perspective. Using the newly developed crosswalks between the legacy measures of SQLI and SRS-22r can facilitate inclusion of PRO data in long-term assessments of treatments for patients with AIS.

Based on linear equating, we provide crosswalk tables that link SQLI scores with SRS-22r scores for the Function, Pain, Self-Image, and Mental Health domains and represents the first linking of SQLI domains with SRS-22r domains. While these crosswalk tables should be considered specific to our population of adolescents with AIS being managed at a pediatric orthopedic facility, this population is likely representative of the larger population of patients with AIS.

We tested standard linking assumptions: high and similar reliabilities, high correlation between scores to be linked, and invariance of linking across subgroups. Domains of the SRS-22r and corresponding domains of the SQLI measure the same constructs in much the same way.

We then employed two observed-score linking methods and one IRT-based linking method to determine the best linking technique for our data based on application on a hold-out validation sample. The observed-score methods resulted in similar results, with linear equating performing marginally better on each of the four domains.

Correlations between SRS-22r domain scores and linked SQLI domain scores in the hold-out validation sample were high, except for the function domain in which case they were moderate. Bias in scores between SRS-22r domain scores and SQLI domain scores were nearly zero for the pain, self-image, and mental health domains; bias was moderate for the function domain. Overall, quality of linking was excellent for all domains except function, in which case quality was acceptable. This is not surprising given the questionable psychometric qualities of this domain [19]. 

The two instruments measure a similar range of the subdomain constructs; maximum scores on SQLI domains correspond closely to maximum scores on SRS-22r domains, and minimum scores on SQLI domains correspond closely to minimum scores on SRS-22r domains (possibly excepting Function). These results indicate the two instruments effectively measure the same scope for each domain. However, due to insufficient numbers of very low scores (< 0.1% of the sample), we were unable to provide correspondences for SQLI domain scores of 0 in the back pain, self-esteem, and moods & feelings domains or a score of 1 in the moods & feeling domain. Care should be taken when using the concordance table for low domain scores, as standard errors of equating are notably higher for approximately the lowest 10% of scores. Conversely, the several domains have moderate ceiling effects are SRS-22 function and pain [19]. While this is not generally problematic as the rate of maximum scores is usually similar for both measures, the SRS-22r subdomain of mental health has notably higher (14.7%) rate of maximal scores than the SQLI subdomain of moods and feelings (7.7%). As a result, the maximum linked score on the crosswalk table for mental health (25.767) is higher than the maximum possible mental health score; this reflects that the SQLI moods and feelings domain is better able to distinguish people with high mental health trait scores.

The crosswalk table generated herein have significant value for AIS researchers interested in quality of life. While use of this table would not be appropriate for making inferences about individual patients, patient scores can be linked and then used in aggregate analyses such as computing mean scores or correlations. Furthermore, because the linking functions employed in Table 3 are linear, sample mean and standard deviations can be easily transformed from SQLI to SRS-22r domain metrics. This allows for translating certain results of published literature using the SQLI into comparable results using the SRS-22r. These translations can then be used for review work, including meta-analysis.

While we do not provide a crosswalk table from the SQLI global score to SRS-22r total scores, researchers interested in a composite overall quality of life score, the linked scores in each subdomain could be combined into a multidimensional composite. As discussed previously, the total score for the SRS-22r may not reflect overall quality of life [19]. 

### Strengths and limitations

Major strengths of this study include use of the single group design, which is the strongest of the linking designs; [40] use of a hold-out sample to validate crosswalk tables; use of a variety of linking strategies including IRT-based and non-IRT methods; and the large sample size. Major weaknesses include a fairly restrictive sample population, limiting generalizability. Additionally, the sample size was insufficient to test invariance of linking across several relevant grouping variables, including race, ethnicity, age, curve magnitude severity, and treatment status (bracing, surgery).

## Conclusion

Longitudinal research that includes PRO scores is critical to allow clinicians to better monitor post-treatment trajectories, predict outcomes, and provide timely interventions that address both physical and psychological dimensions of care of patients with AIS. This study establishes the necessary crosswalks from historical SQLI data to SRS-22r to allow for inclusion in longitudinal studies. The overall quality of linking SQLI and SRS-22r domains is adequate except for function which was moderate. When used in aggregate, transformed SQLI domain scores can safely be used as corresponding SRS-22r domain scores. These findings will allow historical data to be used to assess long-term outcomes of AIS treatments in a large historical sample providing valuable information related to treatment outcomes. Long-term, high-quality PRO research is critical for moving scoliosis management toward a more holistic, patient-centered model. This study expands the ability to include PRO scores, alongside clinical measures, in future long-term follow-up studies providing a more comprehensive understanding of functional and psychosocial impacts of treatments, which could ultimately enhance the long-term management of scoliosis.

## Data Availability

The datasets generated and/or analyzed during the current study are not publicly available due to institutional and IRB policies. The corresponding author can be contacted if you have any questions about the datasets.

## Abbreviations

AIS: Adolescent Idiopathic Scoliosis
PROs: Patient reported outcome measures
SQLI: Scoliosis Quality of Life Index
SRS-22r: Scoliosis Research Society
CFA: Confirmatory factor analysis
BIC: Bayesian Information Criteria
REMSD: Root expected mean square difference
IRT: Item response theory
